# Improving pregnancy outcomes in patients with recurrent implantation failure: The power of RNA-seq-based endometrial receptivity testing

**DOI:** 10.1097/MD.0000000000040210

**Published:** 2024-10-25

**Authors:** Jie Li, Yan Liu, Lin Li, Weijun Chen, Dujuan Xu, Aimei Xiao, Ling Ma, Wanxue Jiang, Lijuan Yang

**Affiliations:** a The Reproductive Hospital of Jiangxi University of Traditional Chinese Medicine, Nanchang, China; b Jiangxi Provincial Key Laboratory of Traditional Chinese Medicine Infertility; c Jiangxi Baijia Emma Obstetrics and Gynecology Hospital, Nanchang, China.

**Keywords:** frozen-thawed embryo transfer, implantation window, pFET, pregnancy outcomes, recurrent implantation failure, RIF

## Abstract

To evaluate whether RNA-seq-based endometrial receptivity testing (rsERT) can improve pregnancy outcomes in personalized frozen-thawed embryo transfer (pFET) during hormone replacement therapy (HRT) cycles among patients with recurrent implantation failure (RIF). We conducted a retrospective cohort study involving 98 RIF patients undergoing HRT for FET. The experimental group consisted of 58 patients who underwent pFET after rsERT, while the control group included 40 patients who refused rsERT and underwent conventional ET. We recorded and examined the subsequent pregnancy outcomes from all cycles. The results of rsERT revealed that 67.24% of the experimental group were out of the “window of implantation” (WOI), with all cases showing a delay. The HCG-positive rate, implantation rate, and clinical pregnancy rate (CPR) in the experimental group were significantly higher than those in the control group, at 75.86% versus 50.00% (*P* = .030), 56.38% versus 31.43% (*P* = .002), and 68.97% versus 47.50% (*P* = .033), respectively. Our study demonstrated that utilizing rsERT technology to guide pFET in HRT cycles significantly enhances implantation and CPRs in RIF patients. Importantly, our findings confirm the effectiveness of rsERT technology and establish a scientific rationale for personalized reproductive medical interventions.

## 1. Introduction

Assisted reproductive technology (ART) offers individuals experiencing infertility the prospect of achieving pregnancy.^[1]^ Nonetheless, approximately 15% of patients experience recurrent implantation failure (RIF) following in vitro fertilization and embryo transfer (IVF-ET).^[2]^ RIF is defined as the failure to achieve an intrauterine gestational sac after 2 or more ET cycles.^[3]^ To address RIF, embryologists have utilized advanced technologies such as time-lapse embryo culture technology (TLT) and preimplantation genetic testing (PGT) to select the most viable embryos for transfer. Moreover, clinicians have designed personalized treatment strategies to counteract other RIF contributing factors, such as immunological disturbances, thrombophilia, and hormonal or metabolic disorders. Despite these efforts, unexplained recurrent implantation failure (uRIF) remains a significant challenge for both patients and physicians due to its unknown etiology.

Successful implantation requires high-quality embryos, endometrial receptivity (ER), and the synchronization of embryo development with the endometrium.^[4]^ ER is the ability of the female endometrium to accept and support embryo implantation during a specific period known as the “window of implantation” (WOI).^[5]^ This period is critical for successful embryo implantation.^[6]^ ER involves various biological and molecular factors, including cellular, morphological, and molecular changes.^[7]^ During the WOI, the endometrial lining undergoes structural changes to create a receptive environment for the embryo.^[8]^ It also produces signaling molecules, such as interleukins, growth factors, and hormones, which interact with the embryo.^[8]^ Accurate assessment of ER is essential in ART and infertility treatment, enabling physicians to determine the optimal timing for ET and thereby increasing the success rate of implantation.

Traditionally, ER evaluation relied on methods such as ultrasound and Doppler blood flow assessment, hysteroscopic detection, endometrial biopsy histology, or the molecular analysis of endometrial aspirated fluid.^[9,10]^ However, these methods have limitations in accurately predicting receptivity status. In recent years, transcriptomic profiling has emerged as a valuable tool for revealing key genes and pathways involved in ER, providing valuable insights into its clinical applications.^[11]^ One such emerging technique is the ER array (ERA),^[12–15]^ a molecular diagnostic test that analyzes the expression levels of 238 genes involved in ER.^[14]^ A study by Ruiz-Alonso et al^[15]^ demonstrated the clinical utility of ERA in identifying personalized WOI and improving pregnancy outcomes for IVF patients. Nevertheless, some researchers argue that ERA is still an unproven technology, and its effectiveness in enhancing implantation success rates remains debatable.^[13]^

RNA-Seq is a next-generation high-throughput sequencing technology for transcriptomic studies. It enables higher resolution differential gene expression analysis compared to whole-genome microarray detection, offering lower detection limits and an unrestricted dynamic range.^[16]^ Professor Li and colleagues have developed an RNA-Seq-based ER test (rsERT) by optimizing experimental design and sampling time combined with machine learning algorithms.^[17]^ This rsERT accurately assesses WOI displacement by analyzing the expression levels of over a hundred relevant genes in the endometrial tissue, including ER-specific marker genes, with high precision and improved reproducibility without relying on morphological changes. Research results demonstrate that personalized ET (pET) guided by rsERT can significantly improve pregnancy outcomes in patients with RIF, highlighting its potential for clinical applications.^[17]^

Patients who cannot ascertain the underlying causes of failed embryo implantation, do not see an improvement in clinical outcomes following clinical interventions, and whose embryos are of high value, exhibit fear towards undergoing ET procedures.. The RNA-Seq-based ER test is a relatively new approach, with only few studies employing this technique to date.^[17,18]^ More research is needed to explore whether pET guided by rsERT can improve pregnancy outcomes in patients with RIF. In the present study, to further verify the potential clinical application of rsERT, we investigated its value for frozen-thawed ET (FET) in patients with unexplained RIF.

## 2. Materials and methods

### 2.1. Study design and patients

This retrospective cohort study was conducted from January 2020 to December 2023 at the Reproductive Hospital of Jiangxi University of Traditional Chinese Medicine. The inclusion criteria were as follows: patients with a body mass index (BMI) of 18 to 25 kg/m², under 35 years of age, a menstrual cycle duration of 24 to 35 days, and a history of uRIF; availability of cryopreserved embryos for FET in hormone replacement therapy (HRT) cycles; normal endometrial thickness on the day before transfer (≥7 mm); normal levels of reproductive hormones (i.e., follicle-stimulating hormone, luteinizing hormone, estradiol, and anti-Müllerian hormone). In this study, uRIF was defined as at least 2 unexplained implantation failures.^[19]^ Patients with abnormal uterine anatomy, intrauterine adhesions, endometriosis, adenomyosis, hydrosalpinx, or uterine fibroids were excluded. A total of 98 patients met the criteria and were included in the study. The patients were divided into 2 groups based on the use of rsERT: the experimental group, consisting of 58 patients who underwent personalized frozen-thawed ET (pFET) after rsERT evaluation, and the control group, comprising 40 patients who declined rsERT and underwent conventional FET in HRT cycles. Baseline demographic characteristics and pregnancy outcomes were collected and analyzed. This study was approved by the Medical Ethics Committee of Nanchang Reproductive Hospital (the Reproductive Hospital of Jiangxi University of Traditional Chinese Medicine) (approval number 2023091), and patient informed consent was waived due to the retrospective nature of the analysis.

### 2.2. Clinical procedures

Endometrial preparation was carried out using an established HRT protocol. HRT was administered prior to endometrial biopsy (in the mock cycle, only for the pFET group) or ET (in the transfer cycle, for all patients). Patients began taking estradiol valerate tablets (3 mg per dose, twice a day; Bayer Healthcare) or red Fematon oral tablets (one tablet 3 times per day, including 2 mg micronized estradiol per tablet; Abbott Healthcare Products B.V.) on the second or third day of menstruation. After 12 days of medication, patients underwent an ultrasonography examination at the hospital. If the endometrial thickness was ≥ 8 mm, luteal support was provided with either progesterone injections (40 mg, twice a day) or vaginal progesterone gel (Crinone gel 8%; Merck Serono SA). Following ET, patients began taking dydrogesterone tablets (20 mg per dose, twice a day). The initial day of progesterone administration was designated as “*P* + 0.” On the 5th day of progesterone administration (*P* + 5) in the mock cycle, uterine curettage was performed to obtain endometrial tissues. Each biopsy sample was stored and sent for rsERT analysis (YK-ERT®) (Yikon Genomics Inc., China) according to the protocol provided by Yikon.

### 2.3. personalized frozen-thawed embryo transfer guided by the RNA-seq-based endometrial receptivity testing /conventional frozen-thawed embryo transfer protocol

In the FET cycle, 1 to 2 embryos were transferred per patient. According to Gardner morphological criteria, embryos with cleavage grades 1 or 2 and 7 to 10 blastomeres were classified as high-quality embryos, while blastocysts graded 4BB or above were considered high-quality. All embryos originated from IVF or intracytoplasmic sperm injection (ICSI) cycles, without PGT cycles, hence the embryos have not undergone genetic testing. In the experimental group, pFET was performed based on the optimal WOI predicted by rsERT. This involved either transferring blastocysts according to the predicted WOI or, alternatively, performing day-3 cleavage-stage ET 2 days earlier. In the control group, patients underwent conventional ET, with frozen-thawed cleavage embryos or blastocysts transferred either on *P* + 3 or *P* + 5. If the endometrium did not reach a minimum thickness of 7 mm on the day before transfer, the cycle was considered for cancelation.

### 2.4. Clinical outcomes

Serum human chorionic gonadotropin (HCG) was measured on the 10th or 12th day after ET. An HCG level ≥ 10 IU was considered positive, and the HCG-positive rate was calculated by dividing the number of HCG-positive patients by the total number of patients. Clinical pregnancy was defined as the presence of at least 1 intrauterine gestational sac on day 28 after ET. The clinical pregnancy rate (CPR) represents the percentage of patients who achieved clinical pregnancy out of the total number of patients. The implantation rate (IR) is calculated as the percentage of successfully implanted embryos among the total number of embryos transferred.

### 2.5. Statistical analysis

Descriptive statistics were used to summarize the general characteristics of the patients. All statistical analyses were performed using SPSS version 26.0 (IBM Corp., Armonk), with a *P*-value < 0.05 considered statistically significant. Continuous variables were presented as mean ± standard deviation or median with interquartile range (IQR), depending on the data distribution. The Mann–Whitney U test or t-test was applied where appropriate. Categorical variables were reported as percentages (counts), and the chi-square test or Fisher exact test was used for comparisons between groups. Binary logistic regression (forward stepwise method) was performed to control for confounding variables.

## 3. Results

### 3.1. Demographics and baseline values

Table 1 details the general clinical demographics and baseline values of patients in the experimental and control groups. There were no statistically significant differences between the 2 groups in terms of age, duration of infertility, infertility type, or the number of previously transferred high-quality embryos. In addition, serum progesterone levels and endometrial thickness on the day before transplantation did not differ significantly between the groups. However, significant differences were observed in BMI (*P* = .032) and the number of ET failures (*P* = .035). The results of rsERT revealed an out-of-WOI rate of 67.24% in the experimental group, with all such cases being delayed by 1 to 2 days. Consequently, embryo transfers were also postponed by 1 to 2 days: cleavage-stage embryos on day *P* + 4 or *P* + 5, and blastocyst-stage embryos on day *P* + 6 or *P* + 7. The median number of high-quality embryos transferred was 2 in both the experimental and control groups. Similarly, no significant differences between the groups regarding the cleavage embryo transfer rate and the blastocyst transfer rate were found.

**Table 1:** Comparison of baseline values and pregnancy outcomes between the experimental and control groups.

Characteristic	Experimental group	Control group	*P*-value
FET cycles, n	58	40	
Age (yr)	30.67 ± 2.87	30.35 ± 3.18	.603
BMI (kg/m^2^)	22.47 ± 2.51	20.92 ± 3.46	.032
Duration of infertility (yr)	3.00 (2.00–5.00)	3.00 (2.00–4.00)	.107
Infertility type, % (n)			
Primary	25.86 (15)	27.50 (11)	.857
Secondary	74.14 (43)	72.50 (29)	
No. of embryo transfer failures (n)	2.00 (1.00–3.00)	2.00 (2.00–3.00)	.035
No. of previously transferred high-quality embryos (n)	3.00 (1.00–5.00)	4.00 (3.00–4.00)	.091
P (nmol/L)	0.34 (0.15–0.59)	0.38 (0.18–0.78)	.374
Endometrial thickness on the day before transfer (mm)	9.00 (8.00–10.00)	9.00 (8.00–10.00)	.249
No. of high-quality embryos transferred, (n)	2.00 (1.00–2.00)	2.00 (1.25–2.00)	.180
Type of transfer embryo, % (n)			
Cleavage	27.59 (16)	35.00 (14)	.434
Blastocyst	72.41 (42)	65.00 (26)	
Out-of-WOI rate, % (n)	67.24 (39)	－	
HCG-positive rate	75.86 (44)	55.00 (22)	0.030
Implantation rate	56.38 (53/94)	31.43 (22/70)	0.002
Clinical pregnancy rate	68.97 (40)	47.50 (19)	0.033

BMI = body mass index, FET = frozen embryo transfer, HCG = human chorionic gonadotropin, P = progesterone, WOI = window of implantation.

Note: Variables are presented as mean ± standard deviation or median with interquartile range unless otherwise specified.

### 3.2. Comparison of pregnancy outcomes between the experimental and control groups

Table 1 shows the comparisons of clinical outcomes between the experimental and control groups. As expected, the HCG-positive rate, IR, and CPR were significantly higher in the experimental group compared to the control group. Specifically, the HCG-positive rate was 75.86% in the experimental group versus 50.00% in the control group (*P* = .030); the IR was 56.38% versus 31.43% (*P* = .002); and the CPR was 68.97% versus 47.50% (*P* = .033).

### 3.3. Multivariate regression analysis of the relationship between confounding factors and clinical pregnancy

Next, a multivariate logistic regression analysis was conducted using variables (BMI and the number of ET failures) that showed statistically significant differences in the univariate analysis to control for potential confounding factors. After adjusting for these confounding variables, pFET following rsERT evaluation still had a positive impact on successful pregnancy (OR = 0.407, 95% confidence interval: 0.177–0.937, *P* = .035) (Table 2).

**Table 2:** Multivariate regression analysis of the relationship between confounding factors and clinical pregnancy.

Variable	β	Standard error	OR	95% CI		*P*-value
				Lower bound	Upper bound	
pFET guided by rsERT	−0.899	0.425	0.407	0.177	0.937	.035

pFET = personalized frozen-thawed embryo transfer, rsERT = RNA-seq-based endometrial receptivity testing.

## 4. Discussion

RIF presents a complex and challenging issue in assisted reproductive therapy. Despite ongoing advancements in medical technology, some patients continue to experience unsuccessful pregnancies after multiple ET attempts. These situations not only diminish patient confidence but also place a significant burden on the healthcare system. The present study aims to explore the potential clinical value of rsERT for RIF patients and to enhance their pregnancy outcomes through rsERT-guided pFET.

Importantly, rsERT is an emerging technology that provides comprehensive insights by analyzing the molecular characteristics of the endometrium, thereby guiding the optimal timing for ET.^[17]^ In a previous study,^[17]^ RIF patients who underwent pET guided by rsERT and received day-3 embryos exhibited significantly higher IPR, IR, and LBR compared to the control group (50.0% vs 23.7%, *P* = .017; 31.4% vs 16.7%, *P* = .033; 42.3% vs 22%, *P* = .056). Similarly, patients who transferred blastocysts also demonstrated higher IPR, LBR, and IR (63.6% vs 40.7%, 59.1% vs 37%, and 43.6% vs 27.3%), though these differences did not reach statistical significance. Pinopodes, a controversial biomarker of ER, have been extensively studied in relation to the WOI and fertility.^[20,21]^ A randomized controlled trial showed that pET utilizing pinopodes significantly improved CPRs for RIF patients compared with conventional FET (33.82% vs 8.11%).^[22]^ Compared to pinopodes, rsERT has been regarded as a superior diagnostic tool for identifying WOI and guiding pET in RIF patients.^[18]^ The rsERT group exhibited significantly higher IPR (50.00% vs 16.67%, *P* = .001), IR (33.33% vs 10.96%, *P* = .001), and LBR (42.86% vs 14.29%, *P* = .004), despite the pinopode group transferring more high-quality embryos. These findings highlight the potential of rsERT in improving the chances of successful embryo implantation.

Our research findings align with previous studies, underscoring the importance of rsERT technology in treating RIF patients. Previous research has demonstrated that rsERT-guided ET can significantly enhance clinical outcomes. These studies support our findings and affirm the effectiveness of rsERT technology. Herein, the IR in the rsERT-guided experimental group was 56.38%, and the CPR was 68.97%, both significantly higher compared to the control group’s IR of 31.43% and CPR of 47.50%.

Given that the rsERT-guided experimental group exhibited a significantly higher BMI and a markedly lower number of ET failures compared to the control group, we conducted a multivariate logistic regression analysis to identify the determinants of clinical outcomes among RIF patients within our study population. The analysis revealed that rsERT-guided pFET significantly enhanced the likelihood of achieving a successful pregnancy in RIF patients (OR = 0.407, 95% CI: 0.177–0.937, *P* = .035). On the other hand, an endometrial biopsy is an unavoidable operation in rsERT analysis. Some researchers posited that endometrial Scratching may enhance embryo implantation by promoting decidualization of the endometrium, eliciting inflammatory responses, and improving synchronization between the endometrium and the embryo.^[23,24]^ However, a large-scale, multicenter, randomized trial conducted prior to IVF showed that endometrial scratching did not lead to improved reproductive outcomes compared to no such intervention.^[25]^ Accordingly, in this study, we believed that identifying the optimal timing for ET using the rsERT was the principal factor contributing to the improved pregnancy outcomes observed in the rsERT-guided experimental group.

Nevertheless, our study has several limitations. Firstly, as with all retrospective observational studies, it is subject to inherent limitations. Secondly, the small sample size could affect the robustness of our findings. Therefore, a more extensive multi-center study is required to further evaluate the broader applicability of rsERT technology and account for the heterogeneity among different patient groups. Additionally, our data focused solely on implantation and CPRs and did not account for obstetric and neonatal outcomes. Thus, we were unable to assess the safety of rsERT-guided pET. A comprehensive prospective cohort study will be crucial in further elucidating the impact of ER testing on personalized ET. Such a study could provide a deeper understanding of the customized approaches facilitated by this testing, enhancing the precision of reproductive interventions. By employing a considerable sample size, we aim to identify subtle patterns and refine personalized strategies, thereby improving the likelihood of successful pregnancies across a broader patient population.

## 5. Conclusions

Our study demonstrated that pFET guided by rsERT significantly enhances implantation and CPRs in HRT cycles among RIF patients. This finding reinforces the feasibility of rsERT technology and provides a solid scientific basis for personalized reproductive medical interventions. The clinical implications and potential applications of rsERT-guided transfer in fertility clinics are considerable. Nonetheless, further research is warranted to validate these findings and refine rsERT-guided transfer techniques for future clinical practice.

## Author contributions

**Conceptualization:** Jie Li, Lijuan Yang.

**Data curation:** Jie Li, Yan Liu, Lin Li, Weijun Chen, Dujuan Xu, Aimei Xiao, Ling Ma, Wanxue Jiang.

**Formal analysis:** Jie Li.

**Investigation:** Jie Li, Lin Li, Weijun Chen, Aimei Xiao, Ling Ma, Wanxue Jiang.

**Methodology:** Jie Li, Yan Liu, Weijun Chen, Lijuan Yang.

**Supervision:** Dujuan Xu, Ling Ma, Wanxue Jiang, Lijuan Yang.

**Writing – original draft:** Jie Li.

**Writing – review & editing:** Jie Li, Lijuan Yang.
